# Color Mapping of Teeth Restored Using Dental Adhesives Loaded with Magnetic Nanoparticles

**DOI:** 10.3390/dj14060333

**Published:** 2026-06-01

**Authors:** Carina-Sonia Neagu, Robert-Angelo Tuce, Rodica Turcu, Izabell Craciunescu, Vlad Mircea Socoliuc, Roxana-Maria Talpos-Niculescu, Luminita-Maria Nica, Virgil-Florin Duma, Cosmin Sinescu

**Affiliations:** 1 Department of Prosthetic Technology and Dental Materials, “Victor Babes” University of Medicine and Pharmacy Timisoara, 9 Revolutiei 1989 Ave., 300070 Timisoara, Romania; carina.neagu@umft.ro (C.-S.N.); sinescu.cosmin@umft.ro (C.S.); 2 Research Center in Dental Medicine Using Conventional and Alternative Technologies, “Victor Babes” University of Medicine and Pharmacy Timisoara, 9 Revolutiei 1989 Ave., 300070 Timisoara, Romania; 3 Department of Functional Sciences, “Victor Babes” University of Medicine and Pharmacy Timisoara, Piata Eftimie Murgu No. 2, 300041 Timisoara, Romania; tuce.robert@gmail.com; 4 National Institute for Research and Development of Isotopic and Molecular Technologies, 67-103 Donat, 400293 Cluj-Napoca, Romania; rodica.turcu14@gmail.com (R.T.); izabella@itim-cj.ro (I.C.); 5 Laboratory of Magnetic Fluids, Center for Fundamental and Advanced Technical Research, Romanian Academy-Timisoara Branch, 24 Mihai Viteazu Ave., 300223 Timisoara, Romania; vlad.socoliuc@academiatm.ro; 6 Research Center for Complex Fluids Systems Engineering, Polytechnic University of Timisoara, Mihai Viteazu Ave. No. 1, 300222 Timisoara, Romania; 7 Odontotherapy-Endodontics, Faculty of Dentistry, “Victor Babes” University of Medicine and Pharmacy Timisoara, 9 Revolutiei 1989 Ave., 300070 Timisoara, Romania; clinci.roxana@umft.ro (R.-M.T.-N.); nica.luminita@umft.ro (L.-M.N.); 8 3OM Optomechatronics Group, Department of Measurements and Electro-Optics, Faculty of Electronics, Telecommunications, and Information Technology, Polytechnic University of Timisoara, 2 Vasile Parvan Ave., 300223 Timisoara, Romania

**Keywords:** dental materials, dental adhesives, magnetic composites, iron oxide nanoparticles, SiO_2_ coating, Ca(OH)_2_ coating, cross-polarization photocolorimetry

## Abstract

**Background and Objectives:** Conventional dental adhesives doped with magnetic nanoparticles (MNPs) hold the promise of preventing microleakages. However, esthetic concerns have motivated the quest for coatings capable of masking the dark color of MNPs. This study aims to quantify regional chromatic differences between teeth restored using dental adhesives with different MNP content. **Materials and Methods:** We prepared cavities in 42 artificial molars and 9 extracted teeth and divided them into 6 groups: Group 0 (G0), G1, and G2, comprising 14 artificial teeth each and G0e, G1e, and G2e, comprising 3 extracted teeth each. In G0 and G0e, we applied the commercial adhesive, in G1 and G1e we applied the adhesive loaded with MNPs with dual coating (SiO_2_ followed by Ca(OH)_2_), whereas in G2 and G2e we applied the adhesive doped with uncoated MNPs. For the statistical analysis of color differences, we employed Bland–Altman plots and the one-sample *t*-test. **Results:** G1 was similar to G0 in terms of color coordinate distribution, whereas G2 was different. Compared to G0, dental fillings from G1 had mean differences of (−0.56, 0.18, −0.07) in the CIELAB color coordinates (L*, a*, b*), respectively, whereas the mean differences between G2 and G0 were (−15.6, −3.5, −15.7). The CIEDE2000 color differences were 1.5 [1.3, 1.6] between G1 and G0 (mean [95% confidence interval]) and 17.0 [16.0, 18.0] between G2 and G0. Nevertheless, 24.4% of the point pairs compared exceeded the acceptability limit for color difference (1.8). **Conclusions:** Although the silica and calcium hydroxide coating is highly effective in alleviating the esthetic impact of MNP-laden dental adhesives, further research is warranted to reduce between-specimen variability.

## 1. Introduction

Magnetic dental adhesives are produced by loading commercially available adhesives with magnetic nanoparticles (MNPs) [1,2]. To this end, iron oxide nanoparticles, such as magnetite (Fe_3_O_4_), are most frequently employed owing to their biocompatibility and superparamagnetism [3].

Magnetically responsive adhesives were first proposed by Li et al. to enable external magnetic manipulation during bonding [1]. Their formulation contained 2 wt% iron oxide MNPs, supplemented with 5 wt% dimethylaminohexadecyl methacrylate (DMAHDM) for antibacterial activity and 20 wt% nano-sized amorphous calcium phosphate (NACP) for remineralization [1]. This composite has increased dentin shear bond strength by over 50%, likely because the magnetic field has enhanced the adhesive penetration into dentinal tubules prior to light curing. Such results have suggested a potential for secondary caries prevention [1].

The effect of magnetic fields on adhesive layer morphology has been characterized by Zaharia et al. [4], who demonstrated that exposure to a permanent magnet before and during photopolymerization reduced adhesive layer thickness by drawing the material into surface irregularities of the prepared dentin. Optical microscopy, scanning electron microscopy (SEM), and micro-computed tomography (CT) confirmed improved interfacial adaptation and reduced susceptibility to microleakage [4].

Garcia et al. subsequently optimized the composition of their MNP-doped adhesive, evaluating its mechanical properties, mechanism of action, and biocompatibility [5]. The adhesive was applied to freshly cut coronal dentin positioned within the inhomogeneous field of a neodymium cylinder magnet placed beneath the tooth. Simulated pulpal pressure was generated hydrostatically by maintaining a water reservoir 20 cm above the dentin surface. Under these conditions, the magnetic adhesive exhibited a 20% increase in the microtensile bond strength compared to the undoped, commercial adhesive. SEM imaging revealed an enhanced mechanical interlocking between the exposed dentin-bound collagen fibrils and the magnetic adhesive [5].

However, iron oxide nanoparticles are typically black, so their incorporation may darken the adhesive and compromise the color of the entire restoration [5]. Indeed, poor esthetics has been reported as a limitation of MNP-reinforced adhesive formulations [4]. This limitation is clinically relevant, as dental appearance strongly influences social perception. Digital image manipulation studies have shown that improved dental esthetics is associated with more favorable judgments of personality traits and attractiveness [6,7]. Consequently, the esthetic impact of magnetic dental adhesives warrants a systematic evaluation.

In a previous study, we assessed changes in restoration color caused by three magnetic dental adhesive formulations [8]. They were prepared by loading a commercial adhesive with iron oxide MNPs without coating, with silica (SiO_2_) coating, or with a dual coating made of a silica layer covered by a sheet of calcium hydroxide (Ca(OH)_2_). As a control, we prepared similar restorations using the undoped adhesive. The overall color differences between the control group and the group of teeth prepared using the dual-coated MNP-doped adhesive were within the 50:50% acceptability threshold (AT) [9] for both the Euclidean ($\Delta E_{ab}$) and the CIEDE2000 formula ($\Delta E_{00}$) [10,11]. In contrast, the adhesive loaded with bare or silica-coated MNPs altered the restoration color beyond the AT [8]. These results align well with those of spectrophotometric measurements of the corresponding nanocomposite powders. The doubly coated magnetite clusters were closest in color to a standard dental shade [12,13], although still larger than the AT expressed in terms of the Euclidean distance, $\Delta E_{ab}$ = 3.5 [14]. Nevertheless, individual differences between the colors of restorations prepared using a commercial dental adhesive and the same adhesive doped with dual-coated particles often exceeded the AT, as revealed by a Bland–Altman analysis—the limits of agreement were [−16.6, 11.9] for $L^{*}$, [−1.25, 1.29] for $a^{*}$, and [−8.4, 4.4] for $b^{*}$ [8].

The question arises whether the inter-individual color differences mainly originate from between-specimen variation or from the spatial chromatic inhomogeneity of each specimen. Indeed, the latter could have caused errors since the restoration color was determined as the mean value of color coordinates recorded in 10 successive measurements taken at various points of the specimen. Nevertheless, the dental spectrophotometer’s sensor utilized in [8] was comparable in area to the restoration, so it could not characterize the color distribution.

Therefore, in a preliminary study [15], we utilized cross-polarization photography to investigate color differences between the adhesive layer and the bulk of the restoration. In this method [16], specular reflection (surface glare) is suppressed by a pair of linear polarizing filters. One of them covers the flash, whereas the other one covers the camera’s macro-objective. Their axes of transmission are mutually perpendicular. Specular reflection preserves the state of polarization of the incident light if it is polarized perpendicularly to the plane of incidence. Therefore, most of the light originating from surface reflections is blocked by the polarizer filter mounted in front of the camera lens. In contrast, the light that undergoes diffuse reflection (on rough superficial or subsurface structures) becomes unpolarized. In consequence, part of it passes through the polarizer filter that covers the objective (see, e.g., the Supplementary Materials, Figure S1). For color assessments, the pictures should also include a gray reference card that enables one to set the white balance in the image analysis software. The results of [15] have suggested that, on average, chromatic differences between the adhesive layer and the composite filling are within the AT for both the conventional adhesive and the same loaded with dual-coated MNPs. Nevertheless, despite the adjusted white balance, the lack of exposure correction precluded comparisons between teeth from different experimental groups. The present study aims to cover this gap.

This study employs standardized cross-polarization digital photocolorimetry (PCM), known as the eLABor_aid (eLAB) system [16,17,18]. It is utilized to measure regional differences between the colors of teeth restored with a commercial adhesive (i) as purchased, (ii) doped with dual-coated MNPs, and (iii) doped with uncoated MNPs. The analysis of the corresponding CIELAB color space coordinates [10] was performed to evaluate our working hypothesis that the incorporation of dual-coated MNPs has a clinically acceptable impact on the color of the composite filling.

## 2. Materials and Methods

### 2.1. Sample Preparation

Experimental specimens were prepared according to the protocol described and illustrated in [8]. In brief, we first prepared similar class I occlusal cavities in 42 molar phantoms and 9 extracted teeth utilizing the high-speed instrument with a cylindrical bur and water cooling. Then, we divided each set into three groups based on the type of dental adhesive used during restoration. Groups 0, 1, and 2 (G0, G1, and G2) comprised 14 artificial teeth each, whereas Groups 0e, 1e, and 2e (G0e, G1e, and G2e) comprised 3 extracted teeth each. Hereafter, the index “e” is employed to designate groups of specimens fabricated from extracted teeth. The same index is used to differentiate color space coordinates of restorations of extracted teeth from color space coordinates of specimens manufactured from artificial molar phantoms.

The control groups, G0 and G0e, consisted of teeth restored by employing Adper Single Bond 2 (3M ESPE, Two Harbors, MN, USA), which is a commercially available dental adhesive that was chosen because it already incorporates silica nanoparticles. The specimens from the experimental groups, G1 and G1e, were processed with the same adhesive doped with dual-coated MNPs (i.e., magnetite nanoparticle clusters wrapped in silica and covered by a calcium hydroxide layer). The negative control groups, G2 and G2e, were restored using the same adhesive doped with bare MNPs.

Both magnetic dental adhesives were obtained via volumetric dosing within reach in any dental practice, by adding one curette of MNP powder to one drop of commercial adhesive and homogenizing the mixture for 15 s by manual stirring. To determine the mass percentage of the MNPs in the magnetic adhesive, we weighed five curettes of each type of MNPs and five drops of adhesive using a Sartorius BP 221 S balance (Sartorius A.G., Göttingen, Germany) with a precision of 0.1 mg and a measurement range of 220 g. Five independent measurements yielded a mass percent concentration of 2.27 ± 0.15% for the adhesive loaded with dual-coated MNPs and 4.16 ± 0.35 for the adhesive loaded with bare MNPs.

**Figure 1:**
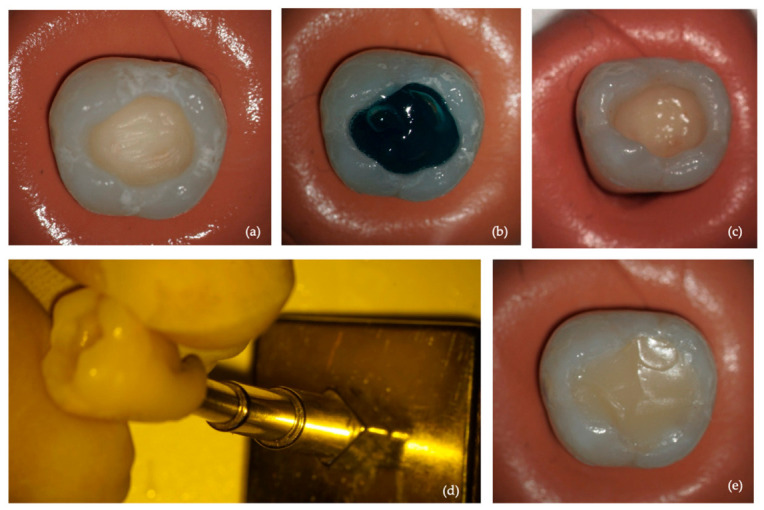
Digital photographs of consecutive stages of specimen fabrication: (**a**) preparation of a class I cavity on the occlusal surface of an extracted tooth; (**b**) application of phosphoric acid etching gel, followed by a 30 s etching period; (**c**) application of the adhesive mixed with dual-coated MNPs; (**d**) 2 min. exposure to the magnetic field generated by a permanent magnet; (**e**) application of the composite resin using the layering technique, followed by light curing.

Specimens from the control groups G0 and G0e were restored as follows: (i) etching gel was placed in the cavity for 30 s (Figure 1b), followed by rinsing and drying; (ii) the commercial adhesive was applied and brushed over the cavity’s surface for 15 s, and compressed air was blown over it for 5 s to render the layer uniform (Figure 1c); (iii) the adhesive was photopolymerized for 10 s; (iv) Filtek Ultimate (3M ESPE Two Harbors, MN, USA) dental composite was added in layers of about 1 mm in thickness and exposed to the curing lamp for 20 s; this step was repeated to fill the entire cavity (Figure 1e); (v) the top of the restored specimen’s crown was removed (specifically, it was cut under running water, at 2 mm below the cuspids); and (vi) the surface was finished using 220 and 400 grit sandpaper, and wet-polished using 1000 grit sandpaper.

We treated the specimens from the experimental groups (i.e., G1, G2, G1e, and G2e) in the same manner, with a single exception: between steps (ii) and (iii), we placed a permanent magnet right below the tooth’s root for 2 min (Figure 1d).

### 2.2. Stereo Microscopy

We first embedded the roots of the specimens in Express STD Vinyl Polysiloxane impression material putty (3M ESPE, Two Harbors, MN, USA) to ensure that their finished surface was horizontal.

Subsequently, we visualized them with a Leica M205 FA stereo microscope (Leica Microsystems, Wetzlar, Germany) to inspect the adhesive layer and the composite filling. We captured the images with a Leica DFC450 C Digital Microscope Camera (Leica Microsystems, Wetzlar, Germany) that features a 5-megapixel charge-coupled device image sensor. All images were acquired at the same magnification, 10×, using the Leica Application Suite X (LAS X) software version 3.0.14 (Leica Microsystems, Wetzlar, Germany).

### 2.3. Standardized Cross-Polarization Digital Photocolorimetry

Regional tooth shade mapping was performed via the eLAB approach, which consists of a systematic processing of digital images acquired by cross-polarized light photography [16].

We immobilized the samples in dental impression material, as described in the previous section, and took digital pictures of their polished surface with a tripod-mounted Nikon DSLR D5300 camera (Nikon, Tokyo, Japan) equipped with an MK-14EXT flash (Meike, Chengdu, China). The distance from the sample to the camera objective was about 140 mm. The flash and the macro-objective of the camera were covered by polarization filters with mutually perpendicular transmission axes. We set the shutter speed to 1/125 s, the aperture to f 22, the sensor sensitivity to 100 ISO, and the image format to RAW.

The polished specimens were photographed on an achromatic background (an 18% gray reference card) for white balance and brightness (i.e., exposure) correction during the image processing stage.

The acquired images were imported into Adobe Lightroom Classic version 15.1.1 (Adobe Inc., San Jose, CA, USA) for standardization. To this end, we used the <Import> button in “Library” mode. Then, we switched to “Develop” mode and carried out the following steps of image processing: (1) in the “Profile” drop-down menu, we selected the “Camera Standard” profile; (2) right-clicked the “Histogram” window and in the context menu selected “Show Lab Color Values”; (3) picked the White Balance selector tool (a pipette), hovered over a neutral gray area representing the reference card, and clicked on a point away from shadows to set the white balance; (4) activated the exposure correction slider by clicking on “0.00”, moved the cursor over the neutral gray area and adjusted the exposure value until the card’s correct lightness coordinate ($L^{*}$) was displayed in the histogram window; and (5) applied the same settings to all images from the given library by selecting the already adjusted (source) image and the other (target) images, clicking the <Sync> button at the bottom-right corner, checking the specific settings to be applied to the target images (“Treatment & Profile”, “White Balance”, and “Basic Tone”), and clicking “Synchronize”.

We assessed the CIELAB color coordinates ($L^{*},\;a^{*},\;b^{*}$) of the selected points from the adhesive layer, as well as from the composite filling by placing the cursor over the given point and by reading the corresponding color coordinates displayed in the “Histogram” window of Adobe Lightroom Classic, in “Develop” mode.

**Figure 2:**
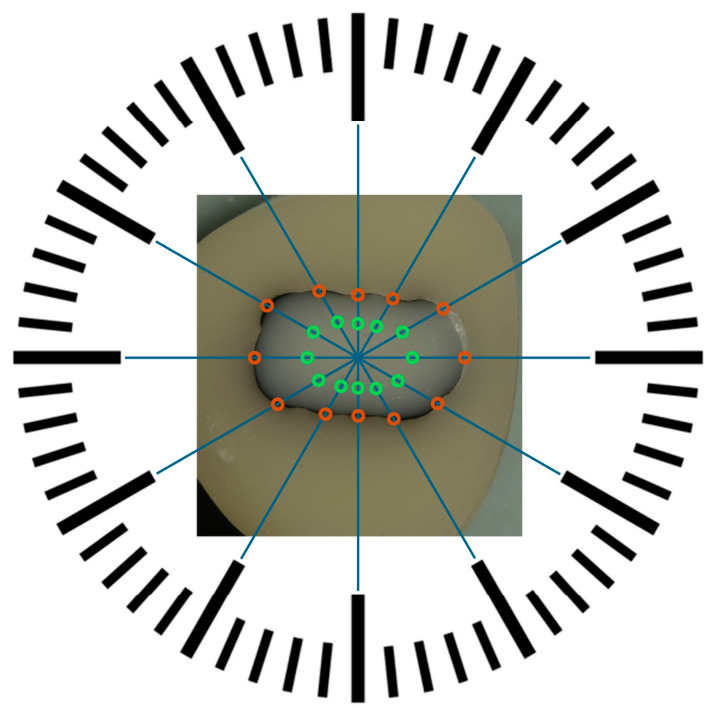
CIELAB color coordinate measurement sites. Within the sketch of a stopwatch dial, there is a representative picture of an artificial tooth specimen from group G2 taken by cross-polarization photography. Orange and green markers indicate the sites of interface and bulk color measurements, respectively.

To avoid the subjective selection of representative points, we recorded the color of the adhesive layer at 12 angular positions corresponding to the hours of the day, from 1 a.m. to 12 a.m. (Figure 2, orange markers). The composite filling’s color was recorded, as well, along the same directions, midway between the adhesive layer and the center of the tooth (Figure 2, green markers).

In this study, the color coordinates of the composite filling are denoted by $L^{*},\;a^{*},\;b^{*}$, whereas those of the interface (adhesive layer and its vicinity) are denoted by ${L^{\prime }}^{*},\;a^{\prime *},\;b^{\prime *}$.

Color differences were quantified in terms of the CIEDE2000 formula:


(1) $$∆E_{00}=\sqrt{{\left(\frac{\Delta L^{\prime }}{K_{L}S_{L}}\right)}^{2}+{\left(\frac{\Delta C^{\prime }}{K_{C}S_{C}}\right)}^{2}+{\left(\frac{\Delta H^{\prime }}{K_{H}S_{H}}\right)}^{2}+R_{T}\left(\frac{\Delta C^{\prime }}{K_{C}S_{C}}\right)\left(\frac{\Delta H^{\prime }}{K_{H}S_{H}}\right)}.$$

The mathematical symbols from this equation were explained in Sharma et al. [19] along with an Excel (Microsoft Inc., Redmond, WA, USA) worksheet designed to compute $\Delta E_{00}$ for pairs of points from the CIELAB color space. Color differences were calculated between the bulks of composite fillings, as well as the adhesive layers, and compared to the AT expressed in terms of the CIEDE2000 color difference, $\Delta E_{00}=1.8$ [20].

### 2.4. Data Analysis

The results of this study are reported in terms of mean ± standard deviation (SD). The statistical analysis of colorimetric data was performed using the MedCalc version 23.4.8 (MedCalc Software, Ostend, Belgium) and the G*Power version 3.1.9.7 (The G*Power Team, Düsseldorf, Germany). The alpha value for statistical significance was set to 0.05 (i.e., a *p*-value less than 0.05 was considered statistically significant).

We relied on the Shapiro–Wilk test to assess the normality of the distribution of experimental data.

To test whether the mean color difference between two experimental groups exceeds the AT, we first computed $\Delta E_{00}$ between pairs of color space points recorded on specimens from the compared groups. Then, we performed a one-sample *t*-test to evaluate the null hypothesis that the mean value of $\Delta E_{00}$ was equal to the AT (i.e., we set the test value to 1.8). If the distribution of color differences deviated from normality, we also conducted a signed rank sum test whose null hypothesis was that the median of $\Delta E_{00}$ is equal to 1.8.

To estimate the sample size, we relied on preliminary data collected on 10 artificial teeth per condition (i.e., subsets of G0, G1, and G2). The mean $\Delta E_{00}$ between 120 pairs of points recorded on the bulk of 10 restorations from G1 and G0 was 1.573, with an SD of 1.166. Sample size was calculated using G*Power for a one-tailed *t*-test [21,22]. The effect size (Cohen’s d) was computed as the difference between the observed mean and the clinically acceptable threshold divided by the SD (i.e., d= (1.8−1.573)/1.166=0.195). Thus, for a statistical power of 0.8 and a significance level of 0.05, the minimum sample size was 165. To exceed it, we recorded the color coordinates at 12 points on 14 artificial teeth.

## 3. Results

Stereo microscopy images of extracted teeth prepared with three different adhesives are shown in Figure S2, in the Supplementary Materials. The restorations created using the magnetic adhesive loaded with dual-coated MNPs (Figure S2b) are hard to distinguish from the control sample (Figure S2a), unlike those doped with bare MNPs (Figure S2c).

**Figure 3:**
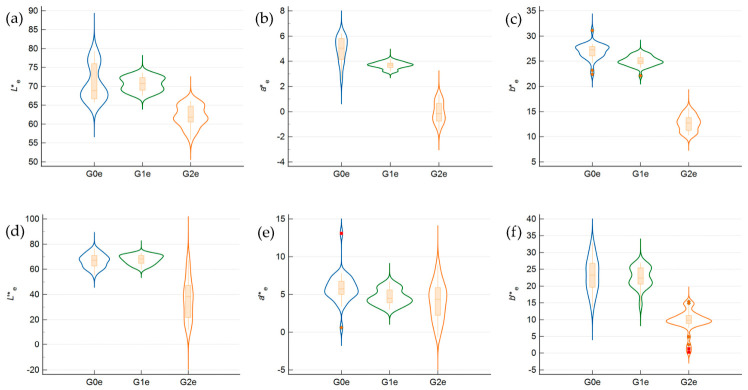
Violin plots of the CIELAB color coordinates of extracted teeth restorations recorded by digital PCM. The plots that characterize the color of the composite filling are shown in terms of (**a**) $L^{*}$ (**b**) $a^{*}$, and (**c**) $b^{*}$, while those that refer to the color of the interface (adhesive layer) are shown in terms of (**d**) $L^{\prime *}$, (**e**) $a^{\prime *}$, and (**f**) $b^{\prime *}$. The index “e” of the color coordinates refers to extracted teeth.

**Figure 4:**
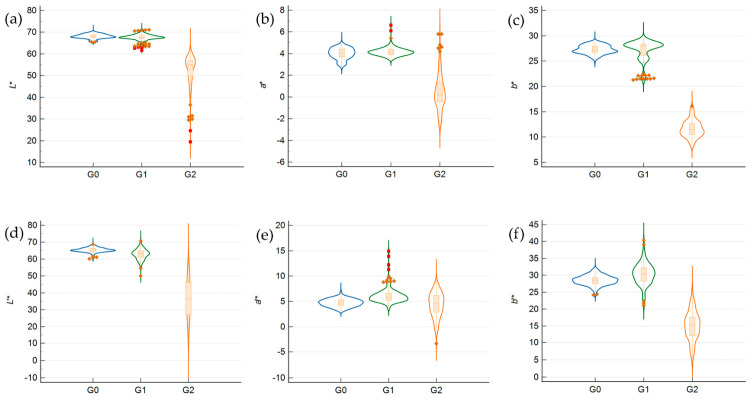
Violin plots of color coordinates of restored artificial teeth. The plots that characterize the color of the composite filling are shown via (**a**) $L^{*}$ (**b**) $a^{*}$, and (**c**) $b^{*}$, while those that refer to the color of the adhesive layer are shown via (**d**) $L^{\prime *}$, (**e**) $a^{\prime *}$, and (**f**) $b^{\prime *}$.

For a quantitative assessment of chromatic differences between the experimental groups, we next performed PCM. The resulting data sets are characterized by violin plots of color coordinates recorded at different regions of extracted teeth (Figure 3) and by molar phantoms (Figure 4).

The results in Figure 3 are consistent with the visual inspection of Figure S2: the medians of color coordinates of G1e are similar to the control, G0e, whereas those of G2e are markedly different, for both the bulk (Figure 3a–c) and the adhesive layer (Figure 3d–f). The sole exception is the redness coordinate of the adhesive layer, $a^{\prime *}$, (Figure 3e). Nevertheless, one can notice differences between the distributions of the color coordinates, characterized by the lateral profile of the violin plots (i.e., plots of the probability density function).

**Figure 5:**
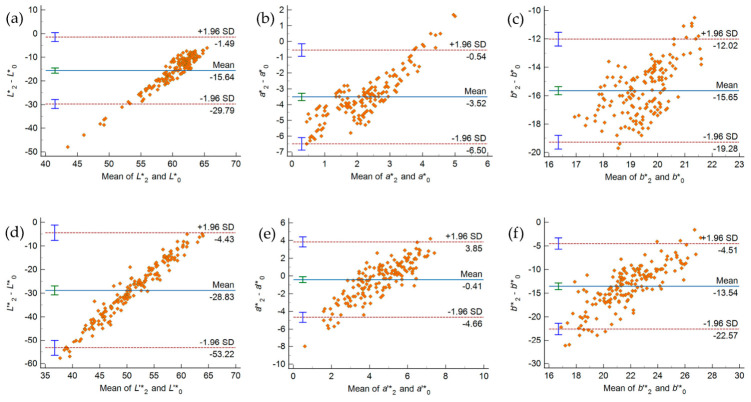
Bland–Altman (BA) analysis of color differences between restorations from G2 and G0. Panels (**a**–**c**) are BA plots of differences vs. means of bulk color coordinates, $L^{*},\;a^{*}$, and $b^{*}$, respectively; panels (**d**–**f**) are BA plots of differences vs. means of the interface color coordinates, $L^{\prime *},\;a^{\prime *}$, and $b^{\prime *}$, respectively.

We next asked the question whether discrepancies between color coordinate distributions stem from the small sample size and/or chromatic differences between the teeth themselves due to biological variability. Therefore, we prepared similar occlusal restorations in 14 artificial teeth (identical molar phantoms) per condition, obtaining the experimental groups G0, G1, and G2. Their PCM analysis provided the results shown in Figure 5.

Figure 4 demonstrates that the restorations created in molar phantoms using the adhesive loaded with dual-coated MNPs (G1) are similar to the control (G0) in what concerns the medians of their color coordinates. Moreover, G1 and G0 have roughly similar color coordinate distributions, but the number of outliers is larger in the case of G1. Just like in the case of extracted teeth, G2 differs from G0 in median color coordinates and their distributions.

To analyze the observed color differences statistically, we utilized the Bland–Altman (BA) method, which is a graphical approach devised for method comparisons [23,24]. The BA plots from Figure 4 and Figure 5 depict differences between the color coordinates of pairs of samples obtained from different groups versus their mean value. The green solid line labeled “Mean” represents the mean difference between the compared samples (also known as bias), whereas the red dotted lines represent the limits of agreement (LoA). The interval of agreement is delimited by the lower limit of agreement (LLA = Mean − SDD) and the upper limit of agreement (ULA = Mean + SDD), where SDD is the standard deviation of the differences. The vertical segments represent the 95% confidence interval (CI) of the corresponding quantities.

Figure 5 indicates that the incorporation of bare MNPs into commercial dental adhesives causes statistically significant changes in most color coordinates of both the bulk of the dental filling (Figure 5a–c) and the adhesive layer (Figure 5d–f). Indeed, for all color coordinates except $a^{\prime *}$, zero lies outside of the corresponding interval of agreement. The lightness coordinate has a remarkably wide interval of agreement, especially in the case of the adhesive layer (Figure 5d). These observations are also valid for the BA analysis of the extracted teeth samples G2e and G0e (Figure S3).

**Figure 6:**
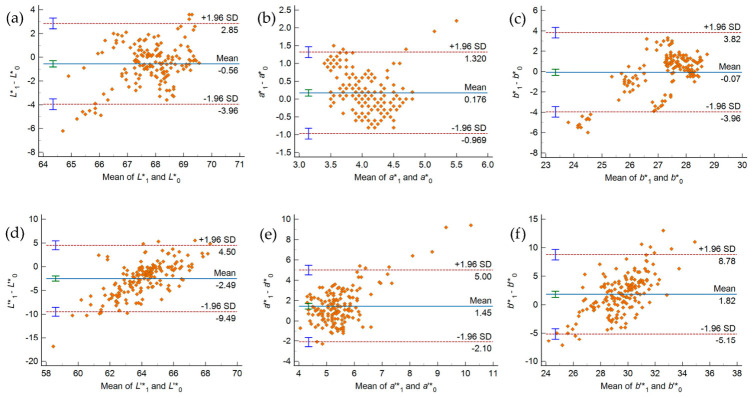
BA analysis of color differences between restorations from G1 and G0. Panels (**a**–**c**) refer to bulk color coordinates, $L^{*},\;a^{*}$, and $b^{*}$, respectively; panels (**d**–**f**) refer to interface color coordinates, $L^{\prime *},\;a^{\prime *}$, and $b^{\prime *}$, respectively.

In contrast, Figure 6 demonstrates that the augmentation of dental adhesives with MNPs coated with both SiO_2_ and Ca(OH)_2_ has a statistically insignificant impact on the color of the restoration: zero lies in the interval of agreement for all the color coordinates. Nevertheless, in the case of the composite filling, the bias is marginally different from zero for $L^{*}$ and $a^{*}$, whereas in the case of the interface it does differ from zero (i.e., its 95% confidence interval does not contain zero). The statistically insignificant impact of dual-coated MNPs on the restoration’s color was reconfirmed by the BA analysis of the extracted teeth groups G1e and G0e, for both the composite filling and the interface (Figure S4). However, unlike in Figure 6, the bias is significantly different from zero for the redness and yellowness coordinates of the composite filling.

To test whether the observed mean color differences between G1 and G0 are clinically acceptable, we computed CIEDE 2000 color differences, $\Delta E_{00}$, between pairs of color space points recorded on samples taken from different groups and applied the one-sample *t*-test. It indicated that $\Delta E_{00}^{\mathrm{Mean}}$ = 1.45 differs significantly from 1.8 (*p* < 10^−4^). Moreover, 1.8 does not belong to the 95% CI of the mean [1.28, 1.61]. However, the validity of the one-sample *t*-test is questionable because the color differences are not normally distributed according to the Shapiro–Wilk test (*p* < 10^−4^), and therefore we conducted the sign rank sum test (which is a non-parametric test). The corresponding *p*-value (*p* < 10^−4^) indicates that the median color difference, $\Delta E_{00}^{\mathrm{Median}}$ = 1.15, with 95% CI [1.00, 1.27], is significantly smaller than 1.8. Nevertheless, 41 out of the 168 point pairs compared exceeded the AT for color difference. When G2 and G0 were compared via the same tests, the mean difference was 16.98, with 95% CI [15.98, 17.97], significantly larger than the AT (*p* < 10^−4^), whereas the median difference was 14.86, with 95% CI [13.97, 15.77], significantly larger than the AT (*p* < 10^−4^). All the point pairs compared exceeded the AT.

**Figure 7:**
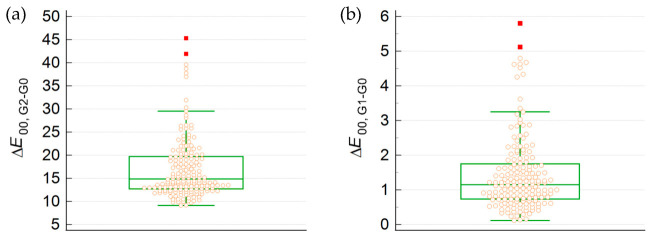
Boxplots of CIEDE 2000 color differences between dental fillings placed in artificial teeth using different dental adhesives. Markers represent color differences between pairs of points from (**a**) G2 and G0, and (**b**) G1 and G0. The box spans the interquartile range, from the first to the third quartile; the horizontal line that divides the box represents the median; the whiskers extend to the points that are at most 1.5 times the interquartile range from the box; points beyond the whiskers are considered outliers; points that are farther than 3 times the interquartile range from the box are termed extreme outliers and are represented by different markers (solid squares).

The boxplots of the color differences between the experimental groups and the control group are shown in Figure 7. They demonstrate how effective dual coating is in reducing the color difference between direct restorations performed by applying the magnetic dental adhesive and the control. Indeed, the median of $\Delta E_{00}$ dropped by more than one order of magnitude due to the MNP coating by silica and calcium hydroxide, as observed by comparing panels a and b of Figure 7.

## 4. Discussion

This study has assessed regional color differences between occlusal restorations performed by utilizing dental adhesives with different MNP contents. Composite fillings placed by using a universal adhesive represented our control groups of extracted teeth (G0e) and artificial molar phantoms (G0). Visual inspection of restored specimens, digital photographs [8] and stereo microscopy images (Figure S2) have suggested that a dental adhesive doped with bare MNPs affects the restoration’s color, whereas one doped with MNPs wrapped in silica and calcium hydroxide does not. This observation motivated our quantitative investigations beyond spectrophotometry, which was unable to characterize color distribution and raised concerns regarding the specimen-to-specimen variability [8].

We employed standardized cross-polarization PCM to estimate CIELAB color space coordinates of the composite filling, as well as of the interface between the tooth and the filling. The latter is a region filled by the adhesive layer. Although the mean color difference between the dental fillings from G1 and G0 have remained well below the AT, $\Delta E_{00}$ = 1.8 [20], certain individual differences exceeded the AT. For example, between-specimens variability led to noticeable differences in lightness, $L^{*}$ (Figure 6a), with even higher discrepancies observed in extracted teeth (Figure S4a). It is not clear at this stage whether the poorer agreement observed in extracted teeth stems from color differences between the teeth, because the interface colors were found in better agreement than the bulks of the direct restorations (Figure S4).

The results reported here did not confirm the hypothesis that the augmentation of a dental adhesive with dual-coated MNPs has a clinically acceptable influence on the color of the direct restoration. Focusing on artificial tooth specimens, this manuscript presents an exploratory, proof-of-concept study; from a clinical perspective, it merely provides encouraging preliminary data. Further studies are warranted on larger samples of extracted teeth to explore the esthetic impact of dental adhesive augmentation with dual-coated MNPs.

Although the magnetic dental adhesive tested in the present study did not meet clinical acceptability standards from the esthetic point of view, it represents considerable progress compared to adhesives loaded with uncoated MNPs. Garcia et al. demonstrated that the addition of bare superparamagnetic iron oxide nanoparticles to the commercial adhesive improved its bonding strength to dentine, but they noticed that the color of the final restoration might be affected and recommended further research [5].

In this study, the chromatic impact of the bare MNP-laden adhesive was illustrated by stereo microscopy (Figure S2) and quantified by PCM. The bare MNP-doped adhesive caused an average drop in $L^{*}$ of 15.6 units in the bulk of the direct restoration and 28.8 units at the interface. By contrast, the addition of doubly coated MNPs reduced $L^{*}$ by 0.6 units in the bulk and 2.5 units at the interface. The dual coating also resulted in large differences in the other color coordinates (compare Figure 4 and Figure 5). In what concerns the mean differences in $L^{*}$ and $a^{*}$, our interface colorimetry aligns well with spectrophotometric assessments of the color of MNP powders: dual coating of iron oxide nanoparticle clusters increased their lightness (${\Delta L}^{*}$ = 28) and their redness (${\Delta a}^{*}$ = 2.7), and reduced their yellowness (${\Delta b}^{*}$ = −1.5) [13]. In contrast, for $b^{*}$ we observed that, compared to the control, the incorporation of uncoated MNPs in the dental adhesive reduced the mean yellowness of the interface by 13.5 units (Figure 5f). In contrast, the doubly coated MNPs had the opposite effect, as they increased $b^{*}$ by 1.8 units (Figure 6f). The origin of this disagreement is not clear. Taken together, these findings indicate that incorporating magnetite clusters in SiO_2_ and covering them with an additional Ca(OH)_2_ shell is an effective strategy for masking their dark color.

Additionally, the dual-coated nanoparticles were found to be highly stable across the typical salivary pH range (4 < pH < 8), and displayed a negative zeta potential, which is known to promote calcium and phosphate deposition on their surface [12].

In a previous work [8], we performed dental spectrophotometry measurements of CIELAB color coordinates of dental fillings placed in artificial teeth. The CIEDE2000 color difference [25] between the mean color coordinates was $\Delta E_{00}$ = 1.88 between the sample involving dual-coated MNPs and the control [8]. This difference is slightly larger than the AT. While this result was encouraging, the BA analysis of the color coordinates revealed large individual differences between pairs of specimens from the best-matching groups (Figure 9d–f of [8]). The most concerning were the lightness coordinates, with a moderate bias, −2.4, but a wide interval of agreement [−16.6, 11.9]. Since 95% of the normally distributed differences are encompassed by the LoAs, the two adhesives could be deemed interchangeable from the chromatic point of view only if the LoAs were small enough to ensure that individual color differences remain below the AT, which was not the case.

We hypothesized that the large variability in lightness differences between restorations from the best-matching groups evaluated by spectrophotometry originated from the uneven color distribution of the tooth specimens. In repeated measurements, the spectrophotometer’s light sensor was placed in different positions, thereby assessing the average color of the underlying materials [8]. Thus, readings performed on inhomogeneous samples could result in scattered data. To test this hypothesis, we relied on an imaging-based approach to assess the CIELAB coordinates at a higher resolution than by using spectrophotometry. The results of the present study support the hypothesis. The BA analysis of cross-polarized photocolorimetry data revealed small lightness differences between dental fillings (bias = −0.6, LoAs = [−4.0, 2.9]) and larger ones between interfaces (bias = −2.5, LoAs = [−9.5, 4.5]). The latter is unsurprising, since the MNPs are in the adhesive, and the MNP-laden adhesive layer is less homogeneous than the composite filling. Remarkably, even for the interface, the width of the interval of agreement obtained by PCM was about twice as small as that observed in average color measurements by using spectrophotometry, underscoring the strengths of standardized cross-polarization PCM.

Finally, it is essential to discuss the limitations of the present study. The first, and most important, is the small size of the extracted tooth samples. Therefore, the statistical quantities that refer to groups G0e, G1e, and G2e should be interpreted with caution. The sample size imbalance between artificial and extracted teeth limits the generalizability of the reported findings.

It must be highlighted that artificial teeth were selected instead of natural teeth in order to minimize variability and ensure a more accurate assessment of the color changes associated with the adhesive system that contains MNPs. Natural teeth possess a complex dentinal structure characterized by the presence of dentinal tubules, which facilitate the penetration of adhesive materials into the dentin substrate. This infiltration process can reduce the thickness of the adhesive layer and consequently alter the perceived color of the material. As a result, the optical measurements may not accurately reflect the intrinsic color of the adhesive formulation. By using artificial teeth, which have a more homogeneous and less permeable structure, the influence of dentinal infiltration is minimized. This allows for a more reliable evaluation of the color variations produced by different types of MNP-doped additives. However, this option reduced the clinical relevance of the current study.

Second, the concentration of MNPs in the dental adhesives has been controlled volumetrically, with limited precision. This approach was chosen to mimic the clinical context, although it most likely contributed to the experimental errors.

Third, the manual mixing of nanoparticle-laden adhesives may have resulted in non-uniform nanoparticle distribution, thereby contributing to the dispersion of the observed color coordinates. Future research may consider using more advanced mixing techniques and a detailed characterization of the nanoparticle distribution within the magnetic adhesive.

Fourth, the resolution of digital photography is insufficient to resolve certain parts of the adhesive layer, of the order of 10 μm to 400 μm in thickness [4]. Therefore, the interface color coordinates do not refer strictly to the adhesive layer but may be influenced by the adjacent materials as well.

Future research is warranted to render the esthetic impact of MNP-laden dental adhesives clinically acceptable. Potential solutions include changes in coating thickness or the application of additional shells, which may also boost the antibacterial properties of the MNPs [26,27]. Besides improvements in the nanotechnological aspects [28,29] of the fabrication of esthetically pleasing magnetic dental adhesives, further developments will also benefit from recent progress in dental shade assessment, which includes the PCM approach employed here [30]. Indeed, a narrative review of the last decade of advancement in dental shade matching demonstrates that, despite advances in technology, it is still challenging to avoid color discrepancies between a restoration and the surrounding teeth [31]. Finally, other variables can also alter the behavior of dental adhesives, such as the type of primer used [32] and the polymerization method [33]. Further studies are needed to characterize the influence of these factors on the performance of MNP-laden dental adhesives.

## 5. Conclusions

This article characterized the spatial distribution of the color of occlusal restorations performed with magnetic dental adhesives.

The results indicate that the incorporation of iron oxide nanoparticles coated with silica and calcium hydroxide into the dental adhesive has a minor but noticeable impact on the color of the final restoration. The mean color shift caused by adding dual-coated nanoparticles to the dental adhesive was smaller than the acceptability threshold in the bulk of the composite filling and close to it at the interface covered by the adhesive layer. However, between-specimen variability resulted in chromatic differences exceeding the acceptability threshold in about a quarter of the compared specimen pairs. Consequently, further validation using larger, clinically representative samples is required.

## Data Availability

The data generated in this study are included in the article and the associated Supplementary Materials. Further inquiries can be addressed to the first author.

## Supplementary Materials

The following supporting information can be downloaded at https://www.mdpi.com/article/10.3390/dj14060333/s1. Figure S1: Digital photographs of restored teeth taken (a) in the absence and (b) in the presence of cross-polarization filters. Both images represent the extracted teeth from G1e, restored using the commercial adhesive doped with doubly coated MNPs; Figure S2: Stereo microscopy images of the experimental samples prepared from extracted teeth. Shown are the specimens from (a) G0e—top row (b) G1e—middle row, and (c) G2e—bottom row. Magnification = 10×. scale bar = 1 mm; Figure S3: BA plots of color differences between restorations from G2e and G0e. Panels (a), (b), and (c) compare the composite filling’s color coordinates, *L*^∗^, *a*^∗^, and *b*^∗^, respectively, whereas panels (d), (e), and (f) compare the interface’s color coordinates, *L*^′∗^, *a*^′∗^, and *b*^′∗^, respectively; Figure S4: BA plots of color differences between restorations from G1e and G0e. Panels (a), (b), and (c) compare the composite filling’s color coordinates, *L*^∗^, *a*^∗^, and *b*^∗^, respectively, whereas panels (d), (e), and (f) compare the interface’s color coordinates, *L*^′∗^, *a*^′∗^, and *b*^′∗^, respectively; Data S1: DataS1-Colorimetric values and ANOVA.xlsx.

## Abbreviations

AT	50:50% acceptability threshold
BA	Bland–Altman
CI	confidence interval
CT	computed tomography
DMAHDM	dimethylaminohexadecyl methacrylate
LLA	lower limit of agreement
LoAs	limits of agreement
MNP	magnetic nanoparticle
NACP	nano-sized amorphous calcium phosphate
PCM	cross-polarization digital photocolorimetry
SEM	scanning electron microscopy
ULA	upper limit of agreement
