# Supplementary material for: Color Mapping of Teeth Restored Using Dental Adhesives Loaded with Magnetic Nanoparticles
Source: Dent J (Basel). 2026 Jun 1;14(6):333. doi: 10.3390/dj14060333 (PMC13298213; doi:10.3390/dj14060333)
Supplement: Supplementary file 1 [file dentistry-14-00333-s001.zip › photocolor_v14_supplementary_material.pdf]

dentistry journal

MDPI

Supplementary Material

Color mapping of teeth restored using dental adhesives loaded with magnetic nanoparticles

Carina Sonia Neagu, Robert-Angelo Tuce, Rodica Turcu, Izabell Craciunescu, Vlad Mircea Socoliuc, Roxana-Maria Talpos-Niculescu, Luminita-Maria Nica, Virgil-Florin Duma, and Cosmin Sinescu

This file provides supplementary information related to the main article entitled "Color mapping of teeth restored using dental adhesives loaded with magnetic nanoparticles". To render it self-consistent, we included detailed figure captions and reiterated the definitions of acronyms and notations used in the article. Nevertheless, to understand the research context and the interpretation of the illustrations presented here, the reader is referred to the main text.

In this study, we used standardized cross-polarization digital photocolorimetry (PCM), to map the colors of natural and artificial teeth restored using a commercial adhesive and novel formulations obtained by doping that adhesive with bare magnetic nanoparticles (MNPs), or the same MNPs wrapped in silica and coated with a layer of calcium hydroxide. We utilized PCM to evaluate the hypothesis that the dual coating effectively masks the dark color of the enclosed iron oxide clusters such that the restoration will suffer clinically acceptable color changes.

Six groups of restored specimens were evaluated: Group 0 (G0) consisted of artificial teeth restored with the conventional adhesive, whereas Group 0e (G0e) consisted of extracted teeth restored with the same adhesive – Adper Single Bond 2 (3M ESPE, Two Harbors, Minnesota); G1 (artificial teeth) and G1e (extracted teeth) were restored using the conventional adhesive loaded with dual-coated MNPs; G2 (artificial teeth) and G2e (extracted teeth) were restored with the conventional adhesive loaded with uncoated MNPs.

In this study, we quantify colors in terms of coordinates of points from the CIELAB color space. Color coordinates of the composite filling's bulk regions are denoted by L^*, a^*, b^*, whereas those of the interface occupied by the adhesive layer are denoted by L'^*, a'^*, b'^*.

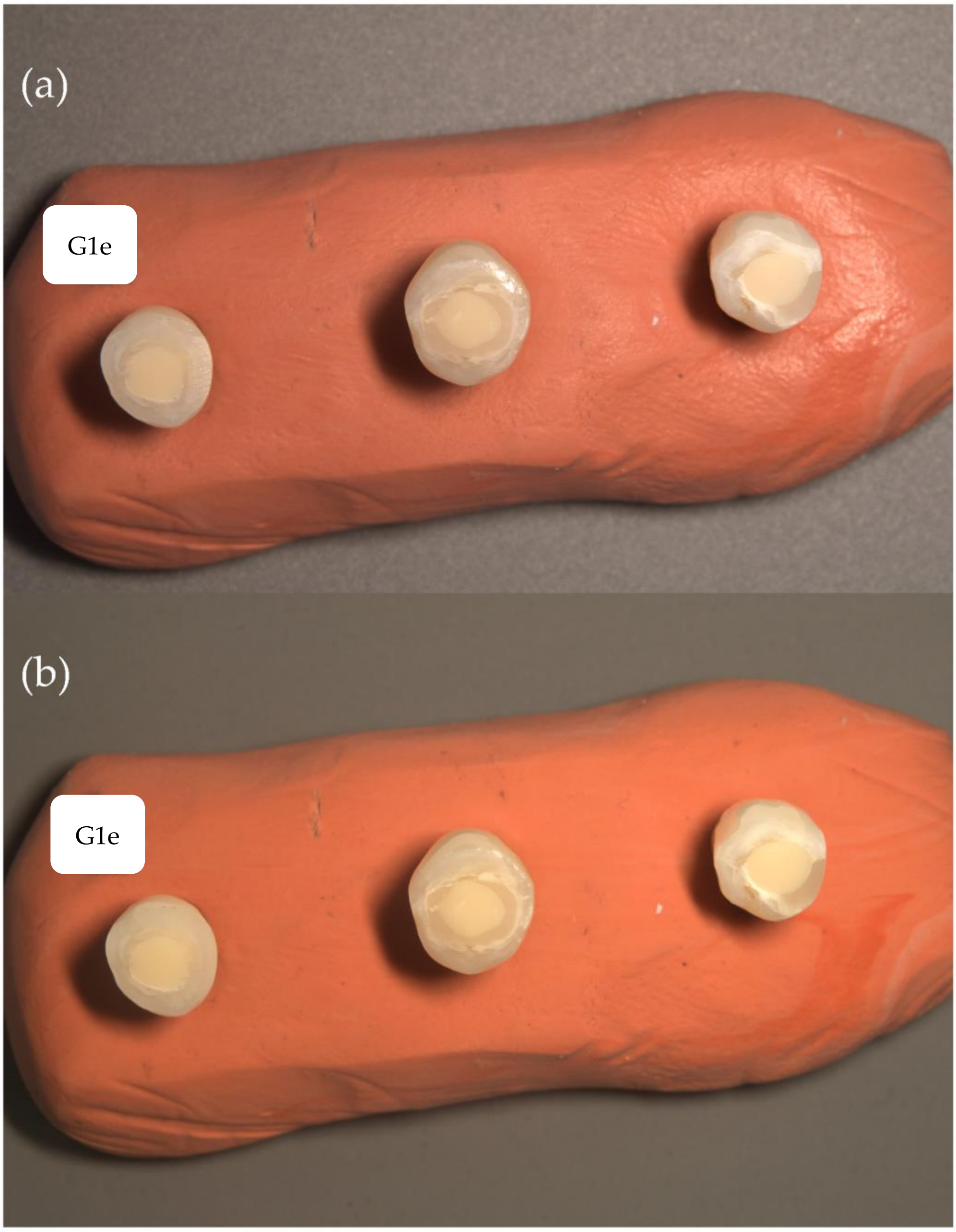

Figure S1. Digital photographs of restored teeth taken (**a**) in the absence and (**b**) in the presence of cross-polarization filters. Both images represent the extracted teeth from G1e, restored using the commercial adhesive doped with doubly coated MNPs.

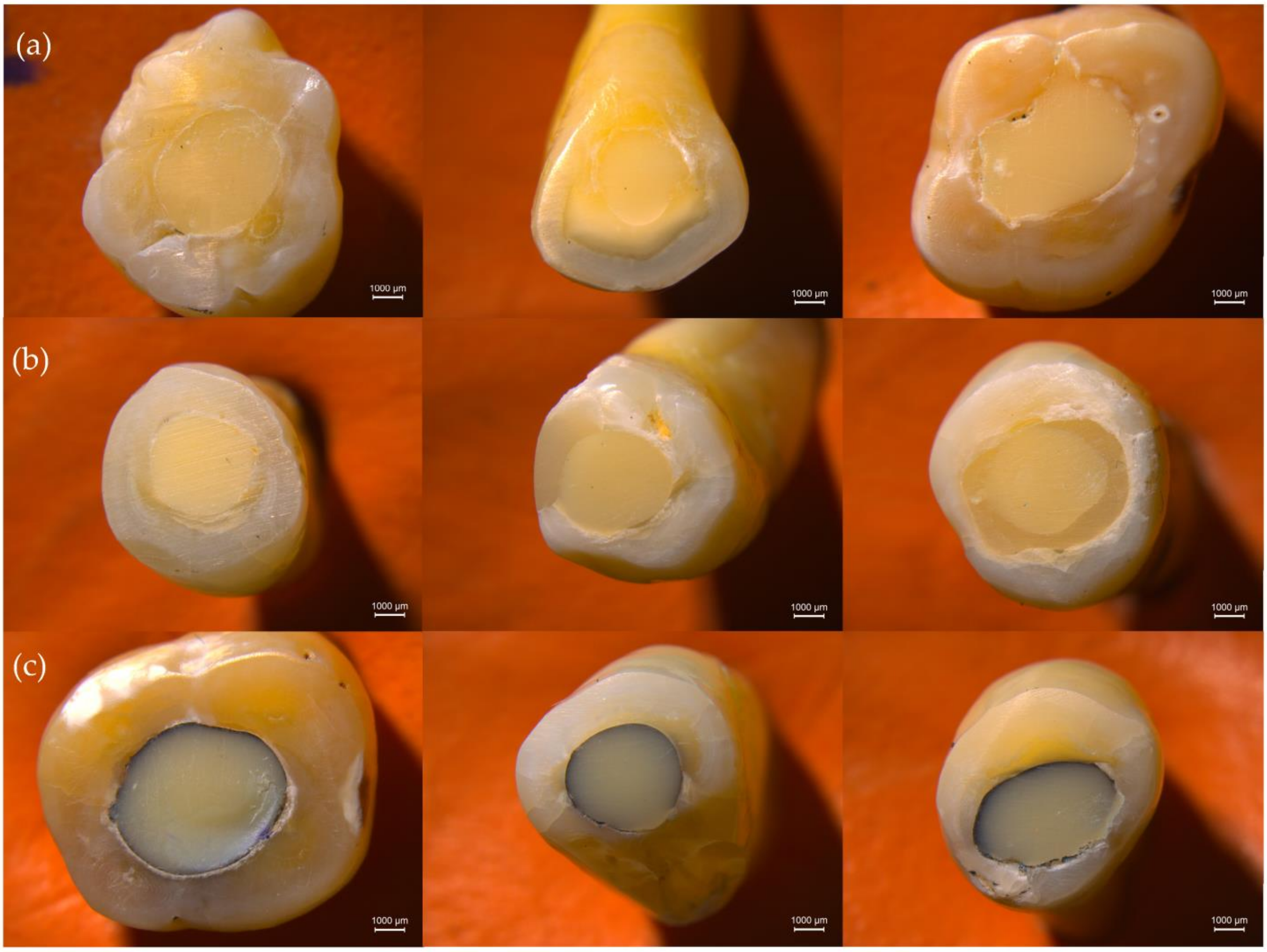

Figure S2. Stereo microscopy images of the experimental samples prepared from extracted teeth. Shown are the specimens from (**a**) G0e – top row (**b**) G1e – middle row, and (**c**) G2e – bottom row. Magnification = 10×. scale bar = 1 mm.

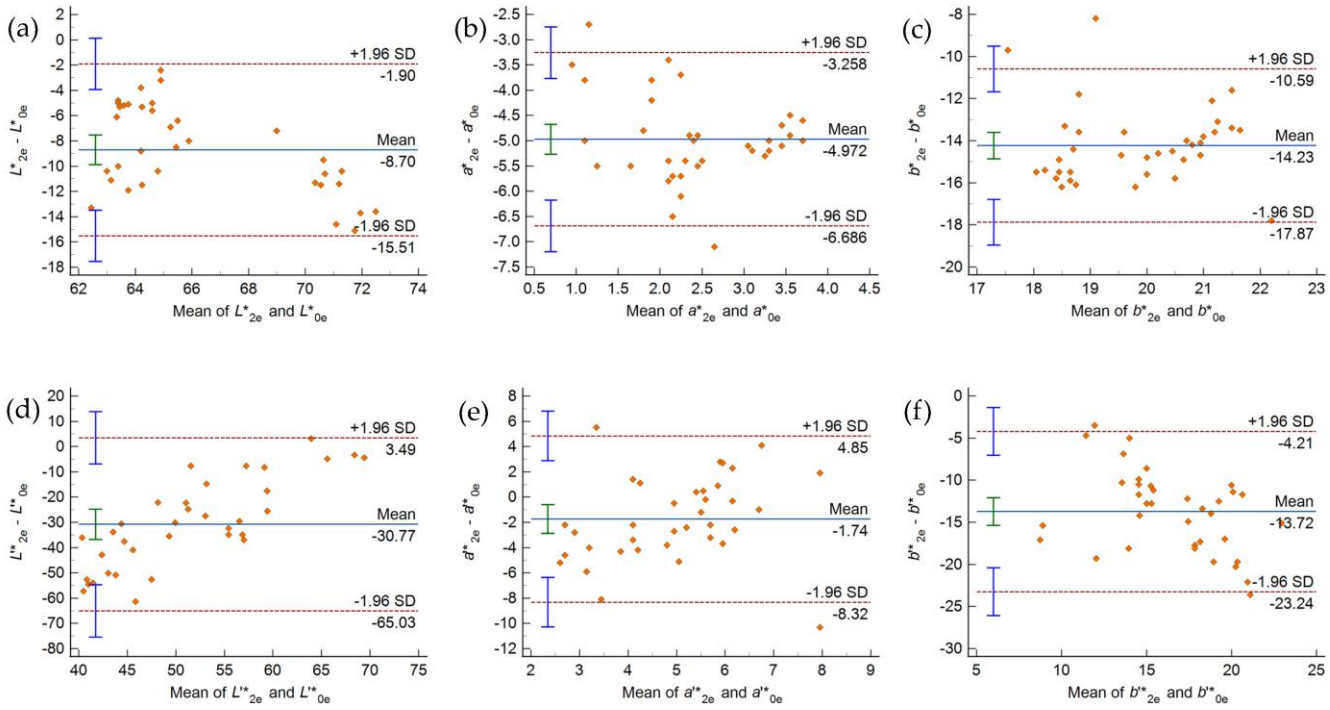

Figure S3. BA plots of color differences between restorations from G2e and G0e. Panels (**a**), (**b**), and (**c**) compare the composite filling's color coordinates, L^*, a^*, and b^*, respectively, whereas panels (**d**), (**e**), and (**f**) compare the interface's color coordinates, L'^*, a'^*, and b'^*, respectively.

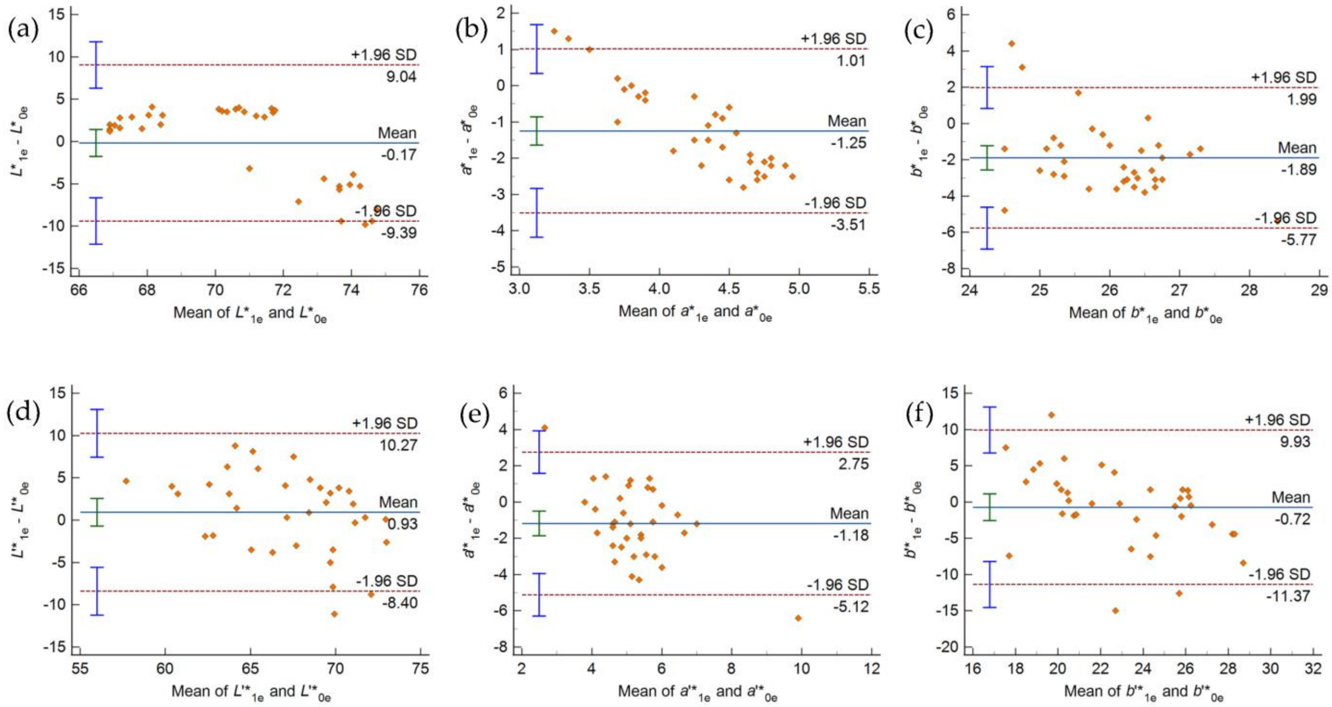

Figure S4. BA plots of color differences between restorations from G1e and G0e. Panels (**a**), (**b**), and (**c**) compare the composite filling's color coordinates, L^*, a^*, and b^*, respectively, whereas panels (**d**), (**e**), and (**f**) compare the interface's color coordinates, L'^*, a'^*, and b'^*, respectively.
